# Successful Removal of Metal Objects Causing Penile Strangulation by a Silk Winding Method

**DOI:** 10.1155/2013/434397

**Published:** 2013-10-31

**Authors:** Chuanjiang Dong, Ziqiang Dong, Fei Xiong, Zonglan Xie, Qiaoli Wen

**Affiliations:** Department of Urology, Yichang Central People's Hospital, The First College of Clinical Medical Science, China Three Gorges University, Yichang 443003, China

## Abstract

Penile strangulation is a challenge to urologists. The decompression of the penis is required to prevent long-term complications. Metal objects are difficult to remove. Cutting is the most common method described. Appropriate cutting tools may be difficult to obtain, and the process may be time-consuming with the possibility of iatrogenic penile injury. In this paper, we will present a simple method to remove such objects by use a silk winding method and subcutaneous puncture.

## 1. Introduction 

Penile strangulation is a rarely described urological emergency. Nonmetallic and thin objects are easy to remove. Metal objects are difficult to remove [1, 2]. Cutting is the most common method described. Appropriate cutting tools may be difficult to obtain, and the process may be time-consuming with the possibility of iatrogenic penile injury [2]. We report two cases of penile strangulation that presented to our emergency department. The patients were treated with a silk winding method and subcutaneous puncture.

## 2. Case Reports

### 2.1. Case 1

**Figure 1:**
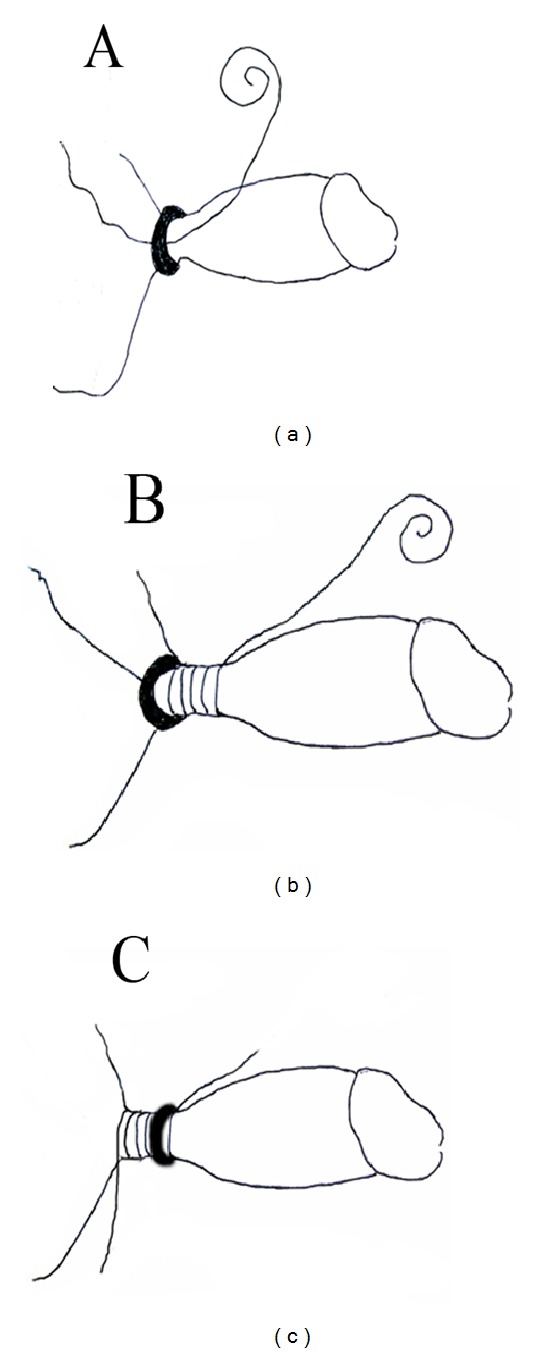
Schematic illustration of the surgical procedure. (a) Ligature of silk string is passed proximally through the metallic ring. (b) The silk was wound around the penis 10 to 15 times just below the metallic ring. Subcutaneous punctures were performed at three sites of the distal part penis foreskin with a 22-gauge needle. A glandular puncture was performed at the glans of penis. (c) The metallic ring is then pushed distally 2 to 3 mm down the penis.

**Figure 2:**
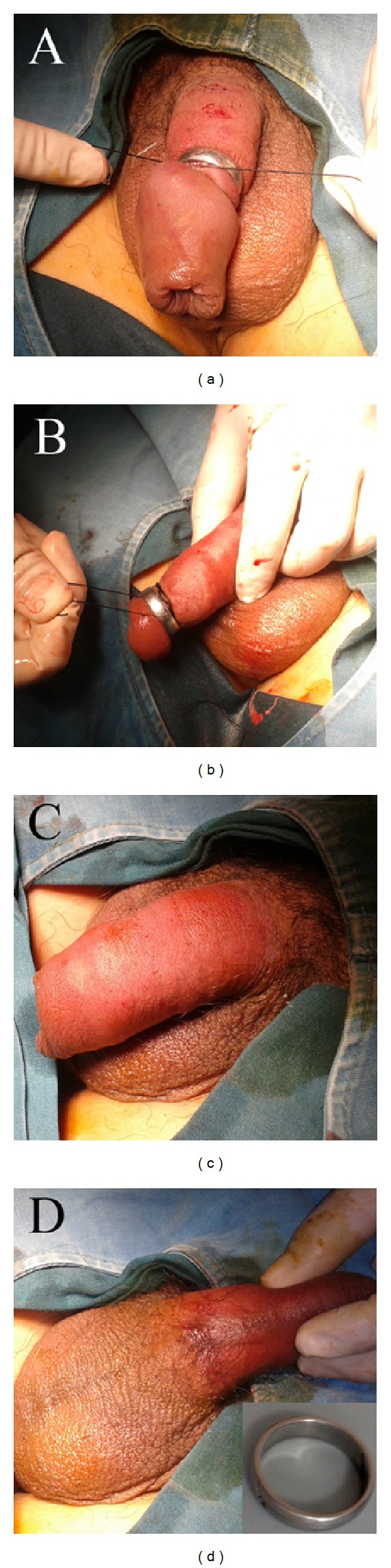
Surgical photograph of the surgical procedure. (a) Ligature of silk string is passed proximally through the metallic ring. (b) The silk was wound around the penis 10 to 15 times just below the metallic ring. The metallic ring is then pushed distally 3 cm down the penis. (c, d) There is no damage to the dorsal and ventral part of the penis. The ring was removed intact from the penis.

A 64-year-old man presented to the emergency department with a metallic ring entrapment on his penis down to the penoscrotal junction for 12 hours. Physical examination revealed a large nontender palpable mass in the lower abdomen, suggesting a distended bladder, extraordinarily engorged edematous penis. We administered 10 mL of 1% lidocaine circumferentially in the penile shaft as local anesthesia. With a 0-0 silk, one end of the silk was passed proximally through the metallic ring. Subcutaneous punctures were performed at three sites of the distal part foreskin with a 22-gauge needle. A glandular puncture was performed at the glans of penis. The penis softened after tissue fluid outflows. The other end of the silk was wound around the penis 10 to 15 times just below the metallic ring. Tissue fluid outflows continuously through the subcutaneous puncture hole during the winding. The proximal side of the silk was unwound from the penis, and the metallic ring was pushed distally 2 to 3 mm to the distal end of the penis. The process of winding and sliding the metallic ring down the compressed area was repeated (Figures 1 and 2). The total operation time was 20 min. The patient was discharged 5 days after the strangulating object was removed. No erection dysfunction was found during the 6-month follow-up period.

### 2.2. Case 2

A 57-year-old male presented to the emergency room with a metallic bearing on his penis which continued for a period of 8 hours. The penis was swollen. Under local anesthesia, the metallic bearing was removed with the method described in Case 1. We just performed subcutaneous punctures at the distal part foreskin; the glandular puncture was not performed because the foreskin was grossly edematous and turgid without blood stasis in the glans of the penis. No erection dysfunction was found during the 6-month follow-up period.

## 3. Discussion

Penile strangulation is a rare injury but requires urgent treatment. In most cases, the act is performed to maintain a longer erection and/or to improve sexual interest [3]. The various case reports illustrated that penile strangulating objects are usually heavy metal rings, hammer-head, and plastic bottle neck, sprockets, or plumbing cuff [2]. In reported cases of penile strangulation, there are many ways to remove the strangulated objects, such as aspiration method, cutting method [4], and deglove operation [5]. Cutting is the most common method described. Cutting tools used included an iron saw, orthopaedic equipment, and a high-speed diamond-tipped dental drill [6, 7]. Cutting process may be time-consuming with the possibility of iatrogenic penile injury [8]. No matter what kinds of strangulated objects they were, we should deal with them from simple to complex and try using less harmless ones. Kwangsung Park described a string method with a glandular puncture, which is easier and quicker than the previous method for removing thick, heavy steel. The glandular puncture is somewhat useful for drainage of ischemic blood in penile strangulation. However, if there was not only congestion of the corpus spongiosum and the glans penis but also the edema of the foreskin, foreskin puncture and glandular puncture should be performed at the same time. If there was only edema of the foreskin without blood stasis in the penis glans, foreskin puncture is enough. Thus, our method represents a further improvement of the method developed by Noh et al. [2].
